# Neurod1 Suppresses Hair Cell Differentiation in Ear Ganglia and Regulates Hair Cell Subtype Development in the Cochlea

**DOI:** 10.1371/journal.pone.0011661

**Published:** 2010-07-22

**Authors:** Israt Jahan, Ning Pan, Jennifer Kersigo, Bernd Fritzsch

**Affiliations:** Department of Biology, University of Iowa, Iowa City, Iowa, United States of America; Texas A&M University, United States of America

## Abstract

**Background:**

At least five bHLH genes regulate cell fate determination and differentiation of sensory neurons, hair cells and supporting cells in the mammalian inner ear. Cross-regulation of *Atoh1* and *Neurog1* results in hair cell changes in *Neurog1* null mice although the nature and mechanism of the cross-regulation has not yet been determined. *Neurod1*, regulated by both *Neurog1* and *Atoh1*, could be the mediator of this cross-regulation.

**Methodology/Principal Findings:**

We used *Tg(Pax2-Cre)* to conditionally delete *Neurod1* in the inner ear. Our data demonstrate for the first time that the absence of *Neurod1* results in formation of hair cells within the inner ear sensory ganglia. Three cell types, neural crest derived Schwann cells and mesenchyme derived fibroblasts (neither expresses *Neurod1*) and inner ear derived neurons (which express *Neurod1*) constitute inner ear ganglia. The most parsimonious explanation is that *Neurod1* suppresses the alternative fate of sensory neurons to develop as hair cells. In the absence of *Neurod1*, *Atoh1* is expressed and differentiates cells within the ganglion into hair cells. We followed up on this effect in ganglia by demonstrating that *Neurod1* also regulates differentiation of subtypes of hair cells in the organ of Corti. We show that in *Neurod1* conditional null mice there is a premature expression of several genes in the apex of the developing cochlea and outer hair cells are transformed into inner hair cells.

**Conclusions/Significance:**

Our data suggest that the long noted cross-regulation of *Atoh1* expression by *Neurog1* might actually be mediated in large part by *Neurod1*. We suggest that *Neurod1* is regulated by both *Neurog1* and *Atoh1* and provides a negative feedback for either gene. Through this and other feedback, *Neurod1* suppresses alternate fates of neurons to differentiate as hair cells and regulates hair cell subtypes.

## Introduction

Neuronal and hair cell development of the inner ear critically depends on the basic Helix-Loop-Helix (bHLH) genes *Neurog1* and *Atoh1*, respectively [1], [2]. However, while several bHLH genes are known in the otocyst, their interplay in prosensory cells to determine neuronal and hair cell differntiation in interaction with other factors remains unclear [3], [4], [5]. Previous work has identified several genes that are co-expressed in both neurosensory primordia in the ear as well as delaminating and differentiating sensory neurons outside the ear, indicating that common upstream regulatory elements may exist for these topologically distinct cells that apparently differentiate into unique adult cells [6], [7], [8], [9]. For example, neurotrophins delineate future sensory areas and are transiently expressed in delaminating neurons that exit the ear adjacent to or overlapping with prosensory regions [10]. Based on this circumstantial evidence it was suggested that these delaminating neurons may have some lineal relationship with the prosensory areas in the ear [6]. If true, ES or iPS cells could be made to develop into both neurons and hair cells, and could regenerate all neurosensory cells lost in deaf patients [11], [12], [13].

This idea of some relationship of neurosensory precursors was further substantiated by studies of two inner ear bHLH genes (*Neurog1*, *Neurod1*). Loss of either gene affects both sensory neuron and hair cells to a variable degree across all epithelia [14], [15], [16]. *Neurog1* is the earlier expressed of the two genes and its absence substantially reduces hair cells in all sensory epithelia [2], [14]. In addition, non-sensory cells such as cells in the cruciate eminence [14], greater epithelial ridge and ductus reuniens [17] are converted into hair cells. In contrast *Neurod1*, which is regulated by *Neurog1* expression in neurons, shows a less profound effect on sensory epithelia [15], [16]. Neurog1 or Neurod1 may have distinct cell-autonomous effect and thus not all cells that are positive for Neurog1 will be positive for Neurod1 which results in some disparity between these two mutations. The simplest explanation for this combined effect on sensory neurons and hair cells by these two bHLH genes is a possible lineage or even clonal relationships of some sensory neuron and hair cell precursors [18], [19]. The observed reduction in hair cells in the respective null mutants could be a consequence of loss of neurosensory precursors [14] or their conversion into hair cells [17]. This idea of lineage relationship is supported by lineage tracing for some neurons and hair cells in mice [20], [21] and the clonal relationship of a small set of neurons and hair cells has been established in chicken [22]. However, technical limitations have thus far precluded establishing unequivocally the degree of this lineage/clonal relationship between all sensory neurons and hair cells.

While these data establish some molecular and in certain cases, lineage and clonal relationship of neurons and hair cells, the molecular basis for the distinct differentiation of either cell type has not been investigated beyond the transcriptional regulation of *Neurog1* and *Atoh1* [20] or short range interactions mediated by delta-notch [23]. This could either happen through *de novo* differentiation of distinct, unspecified otic cells or through successive refinement of cell fate within a given lineage of potentially ambivalent precursor cells. Using a conditional deletion approach we provide here evidence that *Neurod1*, a gene regulated by *Neurog1* in neurons [2] and by *Atoh1* in hair cells [17], suppresses an alternate *Atoh1*-mediated hair cell fate in cells within the ganglia and aids in differentiation of specific hair cell types in the cochlea. *Neurod1* is regulating multiple transcription factors in the neurosensory precursors, which are prematurely expressed in a different pattern in the absence of *Neurod1*. These data provide for the first time a detailed molecular mechanism for cell fate switching in neurosensory precursors of the mammalian inner ear and show that it hinges on suppression of alternate fates in neurosensory precursors by *Neurod1*.

## Materials and Methods

### Ethics Statement

All animal procedures were approved by the University of Iowa Animal Care and Use Committee (IACUC) and conducted according to their guidelines (ACURF #0804066).

### Mice and genotyping for generation of conditional *Neurod1* knockout mice (CKO)

Previously, lethality of newborn *Neurod1* systemic null mice due to severe diabetes arrested the analysis in postnatal mice. To overcome this problem, we extended our analysis in the inner ear using *Neurod1* conditional knockout mice [*Neurod1^f/f^,Tg(Pax2-cre)*]. By generating the *Neurod1* conditional knockout (CKO) mice we could successfully circumvent the effect of *Neurod1* in pancreatic β-cell development and could rescue mice to adulthood in Mendelian ratio.

To generate the *Neurod1* conditional knockout mice we crossed the *Pax2*-cre line [24] with the floxed *Neurod1* line [25]. For this study we used crosses between homozygotic floxed *Neurod1* mice (*Neurod1^f/f^*) with heterozygous *Neurod1^f/+^,Tg(Pax2-cre)* mice. The resulting *Neurod1^f/f^,Tg(Pax2-cre)* mice are conditional knockout (CKO) mutant and the *Neurod1^f/+^,Tg(Pax2-cre)* heterozygous siblings serve as controls, here referred to as wild-types. To show the endogenous *Neurod1* expression by the lacZ reporter, we have used *Neurod1^f/z^,Tg(Pax2-cre)* mice as mutant and *Neurod1^+/z^* mice as control.

We also analyzed the *Neurod1* CKO using *Tg(Atoh1-cre)*. We generated the mice by breeding the homozygous floxed *Neurod1* (*Neurod1^f/f^*) [25] and *Tg(Atoh1-cre)* with a ROSA26 reporter [17] as previously described [26].

Offspring were genotyped by PCR analysis of tail DNA using *Cre*-specific primers which produce a 280 bp product, and *Neurod1*-specific primers which produce a 400 bp product from *Neurod1* coding region and a 600 bp product from the floxed allele. Embryos were collected from timed pregnant females at embryonic day 10.5 (E10.5), E11.5, E12.5, E14.5, E16.5 and E18.5 counting noon of the day the vaginal plug was found as E0.5. We have also analyzed post-natal day 0 (P0), P7, P14, P16 and P30 mice. Pregnant mothers or juvenile mice were anesthetized with a lethal dose of Avertin (1.25% of 2.2.2-tribromoethanol at a dose of 0.025 ml/g of body weight). Embryos were dissected from the uterus and perfused with 4% paraformaldehyde (PFA) in 0.1 M phosphate buffer (pH 7.4) using a peristaltic pump. Heads were isolated and fixed in 4% PFA for further analysis.

### X-gal staining

After perfusion with 4% PFA, mice were hemisected and ears were dissected in 0.4% PFA. After brief washes with phosphate buffer, the samples were stained in a solution containing 0.1 M phosphate buffer, 0.01% deoxycholic acid, 0.02% NP40, 2 mM magnesium chloride, 5 mM potassium ferricyanide, 5 mM potassium ferrocyanide and 0.1 mg/ml X-gal (5-bromo-4-chloro-3-indolyl- β -D-galactoside) for up to 24 hours at room temperature [27].

### *In situ* hybridization

*In situ* hybridization was performed using the RNA probe labeled with digoxigenin. The plasmids containing the cDNAs were used to generate the RNA probe by *in vitro* transcription. The following probes were graciously provided: *Atoh1*; Dr. Zoghbi; *Neurog1*, Dr. Ma; *Fgf8*, Dr. Pirvola; *Pou4f3*, Dr. Xiang; *Nhlh1* and *Nhlh2*, Dr. Braun; *Sox2*, Dr. Cheah. The dissected ears were fixed in 0.4% paraformaldehyde, dehydrated in 100% methanol and rehydrated and then digested briefly with 20 µg/ml of Proteinase K (Ambion, Austin, TX, USA) for 15–20 minutes. Then the samples were hybridized overnight at 60°C to the riboprobe in hybridization solution containing 50% (v/v) formamide, 50% (v/v) 2X saline sodium citrate (Roche) and 6% (w/v) dextran sulphate. After washing off the unbound probe, the samples were incubated overnight with an anti-digoxigenin antibody (Roche Diagnostics GmbH, Mannheim, Germany) conjugated with alkaline phosphatase. After a series of washes, the samples were reacted with nitroblue phosphate/5-bromo, 4-chloro, 3-indolil phosphate (BM purple substrate, Roche Diagnostics, Germany) which is enzymatically converted to a purple colored product. The ears were mounted flat in glycerol and viewed in a Nikon Eclipse 800 microscope using differential interference contrast microscopy and images were captured with Image-Pro software.

### Immunofluorescence

For immunofluorescence staining, the ears were dehydrated in graded ethanol overnight and rehydrated in graded ethanol and PBS. Samples were then blocked with 0.25% normal goat serum in PBS containing 0.01% Triton-X-100 for 1 hour. Then the primary antibodies for Myo VIIa (Myosin VIIa, Proteus Biosciences), Tubulin (Sigma), Caspase 3 (Cell Signaling Technology) and espin (a gift from Dr. J. Bartles JR) were used in dilutions of 1∶200, 1∶800, 1∶100 and 1∶5 respectively and incubated for 24–48 hours at 4°C. After several washes with PBS, corresponding secondary antibodies (1∶500) (Alexa fluor molecular probe 647 or 532 or 488; Invitrogen) were added and incubated overnight at 4°C. The ears were washed with PBS and mounted in glycerol and images were taken with a Leica TCS SP5 confocal microscope.

### Plastic embedding and Stevenel's Blue staining

The end organs of ears were fixed in 2.5% glutaraldehyde overnight followed by several washes with 0.1 M phosphate buffer and then fixed with 1% osmium tetroxide for up to 1 hour. Samples were then washed with deionized water and dehydrated in graded ethanol followed by propylene oxide, embedded with Epon 812 in beam capsules and baked at 60°C for 48 hours. 2 µm sections were cut using a Reichert Ultratome and stained with Stevenel's Blue [28] made of 2% potassium permanganate and 1.3% methylene blue.

For higher resolution and co-localization of probes and proteins, we performed *in situ* hybridization for *Fgf8*, followed by Myo VIIa immunocytochemistry on same ears. Some of these ears were embedded in resin, sectioned and imaged with epifluorescent and transmitted light on a Nikon E800. Some of these sections were counterstained with Stevenel's blue for more detailed histology.

### SEM

P30 mice were lethally anesthetized and perfused with 4% PFA. Ears were dissected, decalcified in EDTA and osmicated [1% OsO4 in 0.1 M phosphate buffer (pH 7.4)]. Osmicated ears were washed several times in distilled water to remove all ions, dehydrated in a graded ethanol, critically point dried, mounted on stubs and coated with gold/palladium. Stubs were viewed with a Hitachi S-3400N Scanning Electron Microscope with 2MeV acceleration.

## Results

### Ectopic hair cells form in sensory ganglia of *Neurod1* conditional null mice

**Figure 1:**
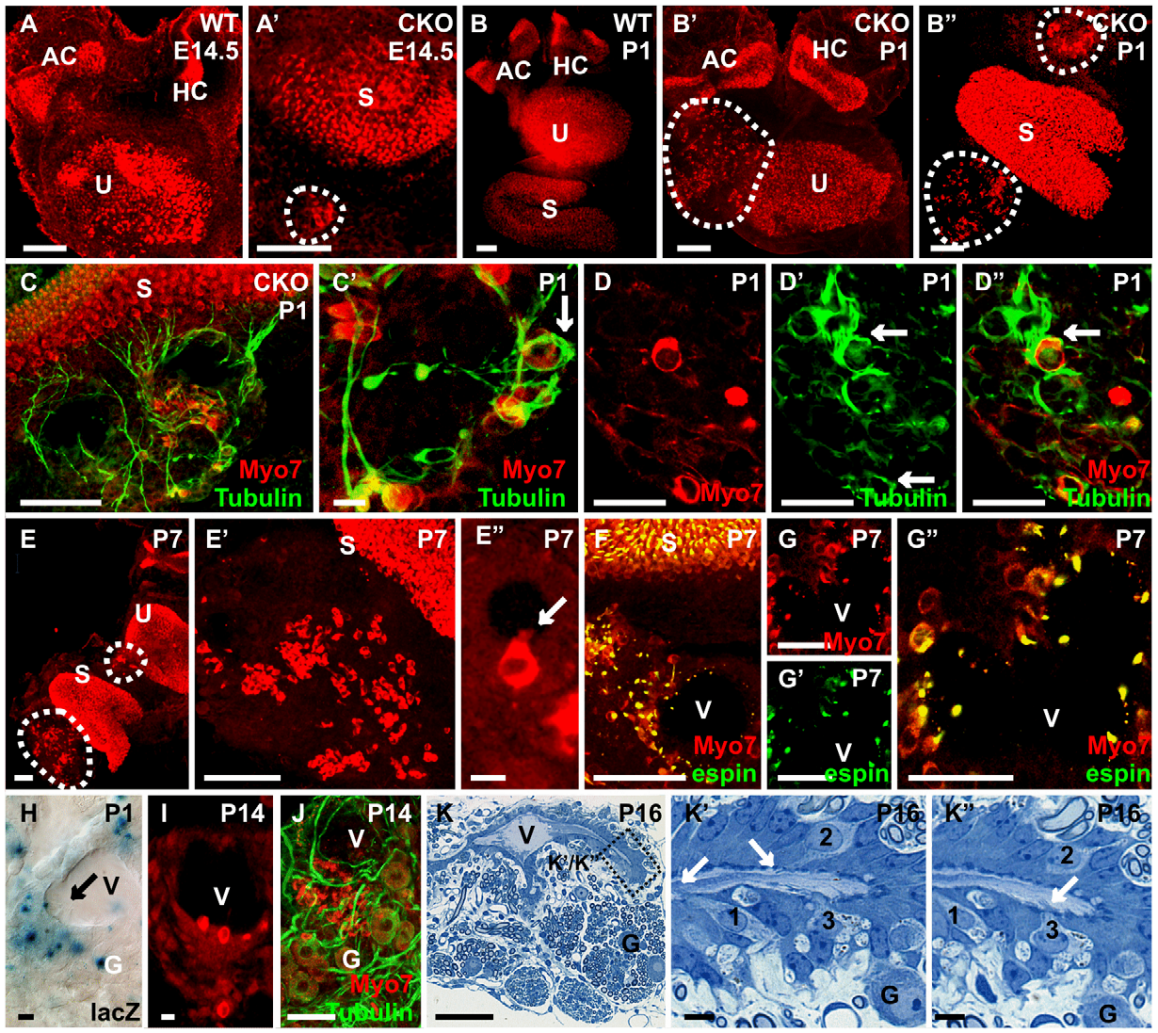
Absence of *Neurod1* results in formation of MyoVIIa positive ectopic hair cells in inner ear ganglia. Whole mount Immunofluorescence labeling with Myo VIIa labels the hair cells in the vestibular epithelia of wild-type mice exclusively inside the sensory epithelia (A,B). In contrast, in *Neurod1* CKO mice, Myo VIIa positive cells are also found outside the sensory epithelia interspersed among the remaining vestibular ganglia near the utricle and the saccule from E14.5 until adulthood (A’, B’, B”, E,E’, I). These Myo VIIa positive cells are often grouped around intraganglionic vesicles (C, E”, F, G, G’, G”, I, J). Immunofluorescence labeling with anti-β-tubulin shows innervation of these Myo VIIa positive cells, some of them apparently form a calyx (arrows in C’, D’, D”). Closer examination shows that some of these cells assume a hair cell like shape with the formation of apical specialization protruding into vesicles (arrows in E”, H). We confirm with anti-espin antibody that these protrusions are stereocilia of these hair cell-like cells (F–G”). Formation of vesicles is also apparent in *Neurod1*-lacZ histochemically reacted ears (H) where β-galactosidase positive cells also protrude stereocilia into the vesicular lumen (arrow in H). These vesicles persist within the vestibular ganglia near the saccule (J). The vesicles are surrounded by cells forming an epithelial layer with hair cells displaying apical specializations protruding into the lumen shown in thin plastic sections (K, K’, K”). I,2,3 in (K’, K”) indicates three ‘intraganglionic hair cells’ in two consecutive sections showing apical protrusions into the vesicular lumen as well as enlarged contacts. AC, anterior canal crista; HC, horizontal canal crista S, saccule; U, utricle; G, ganglion cell; V, intraganglionic vesicle, Bar indicates 100 µm (except 50 µm in G” and10 µm in E”, C’, H and K’, K”).

Previous work has shown that most cochlear and many vestibular sensory neurons are lost in *Neurod1* null mice [15] with the surviving neurons projecting aberrantly to the sensory epithelia of the ear and into the brain [29]. When we studied the detailed distribution of sensory epithelia in whole mounted ears using Myo VIIa as a marker for hair cells to quantify the effects of loss of *Neurod1* on hair cell development, we found numerous Myo VIIa positive cells scattered in the two remaining neuronal aggregations of vestibular and cochlear sensory neurons near the saccule and utricle (Fig. 1A, B’–K’). We also observed the appearance of these Myo VIIa positive cells in ganglia at different stages of *Neurod1* CKO mice as early as E14.5 to near adult (E14.5, P0, P7, P14 and P30; Fig. 1). Many of these cells were grouped around multiple vesicles that were present within these ganglia (Fig. 1H,I). Immunofluorescence labeling with anti-β-tubulin antibody revealed that these cells were densely innervated and occasionally showed formation of a calyx (Fig. 1C’,D’, D”). Some of them had hair like bundles of apical specializations projecting from their apex into these vesicles (Fig. 1E”, I). To confirm that these apical processes were stereocilia, not microvilli, we next performed anti-espin immunofluorescence staining, a marker for stereocilia [30]. We detected espin immunostaining in stereocilia of these cells which, combined with the Myo VIIa marker identified them as hair cells (Fig. 1F–G”). The hair cells around the vesicles could be detected at least until P30 (P16 shown in Fig. 1K–K”) and the vesicles inside the ganglia persisted for about 9 month after birth (data not shown). These cells were surrounded by other cells with luminal contact, had ciliary protrusions into the vesicle and their base showed what appeared to be enlarged synaptic boutons (Fig. 1K’,K”). The cells outlining these vesicles seemed to be in contact with perineurial fibrocytes (Fig. 1K–K”).

In conclusion, our data suggest that *Neurod1* expression in differentiating sensory neurons suppresses the development of Myo VIIa positive hair cells within inner ear ganglia. Alternatively, neural crest derived Schwann cells or mesenchyme derived fibroblasts, cell types that never express *Neurod1*, could be transformed into hair cells through the lack of interaction with *Neurod1* containing neurons. We next tested more definitive markers for hair cell fate acquisition to obtain more insights into this transformation process.

### Gene expression suggests that lack of *Neurod1* transforms some surviving neurons into hair cells

**Figure 2:**
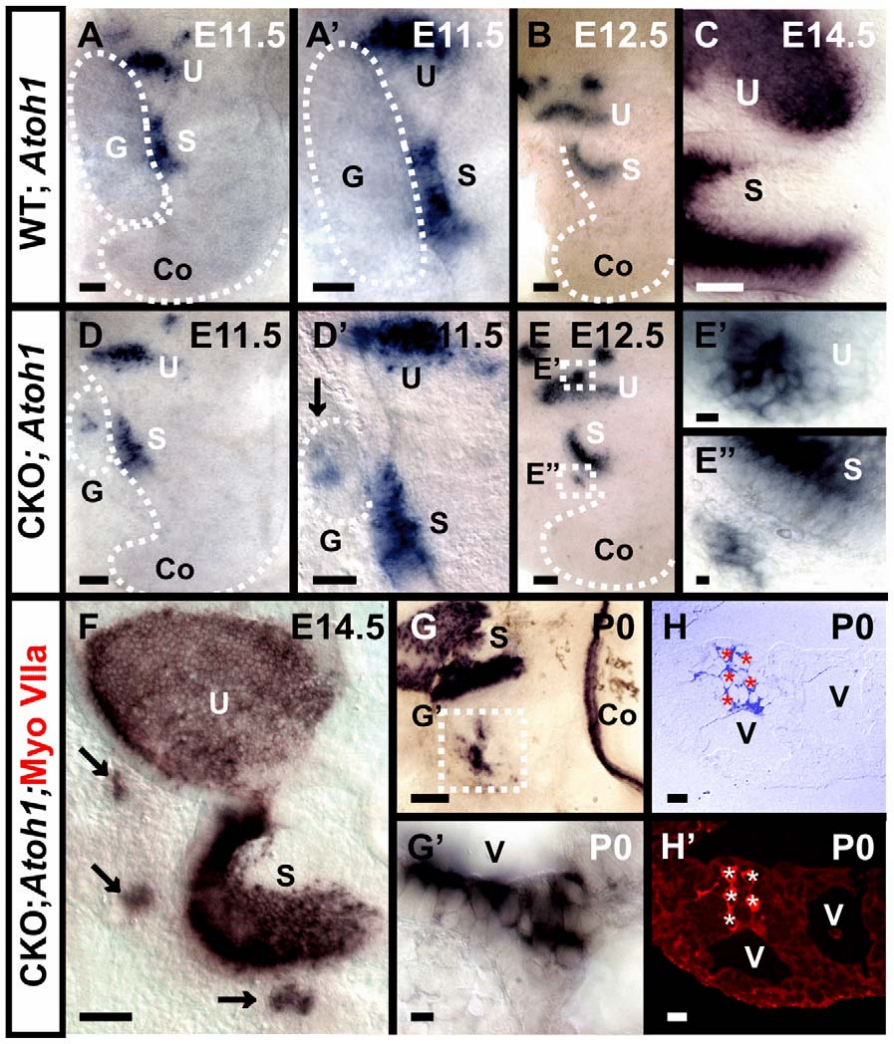
Persistent *Atoh1* expression in remaining ganglia relates to transformation of ganglionic cells into hair cells in *Neurod1* mutant. *In situ* hybridization of *Atoh1* shows a faint and transient expression at E11.5 in some vestibular ganglion cells in wild-type mice (A, A’). This expression is more profound and continues in the ganglia of *Neurod1* CKO mice (D, D’). In later stages, *Atoh1 in situ* signal appears in a cluster of cells in CKO mutants near the utricle and saccule (E–E’, F, G–G’). Some of the *Atoh1* positive cells are aligned along the vesicular lumen similar to the Myo VIIa positive cells shown in Fig. 1 (G). To investigate co-localization, we labeled *Atoh1 in situ* reacted ears with anti-Myo VIIa antibody, embedded in plastic and sectioned. The sections reveal co-localization of Myo VIIa with *Atoh1* in these cells (H,H’) thereby providing strong evidence that these cells are hair cells. S, saccule; U, utricle; G, ganglia; V, intraganglionic vesicle. Bar indicates 100 µm except E, E’ and 10 µm in E, E’.

*Atoh1* is a hair cell differentiation marker with a well established role in the ear only in the differentiation of hair cells [1], [31], [32]. Only limited expression of *Atoh1* has been reported with sophisticated techniques in sensory neurons [17] and none in the neural crest derived Schwann cells or mesenchyme derived perneurial fibrocytes. Using *in situ* hybridization we observed only a transient *Atoh1* expression in the vestibular ganglia of wild-type mice at E11.5 (Fig. 2A, A’). However, in the *Neurod1* CKO mice, *Atoh1* expression continued in these ganglia past the transient expression found in control animals (Fig. 2D, D’). *Atoh1* expression was more profound in later stages and was found in a cluster of cells in the remaining ganglia next to the utricle and saccule, mostly around the ectopic vesicles (Fig. 2E–G’). Immunofluorescence labeling with Myo VIIa antibody revealed that each of the *Atoh1* positive cells was also immunopositive for Myo VIIa (Fig. 2 H,H’).

**Figure 3:**
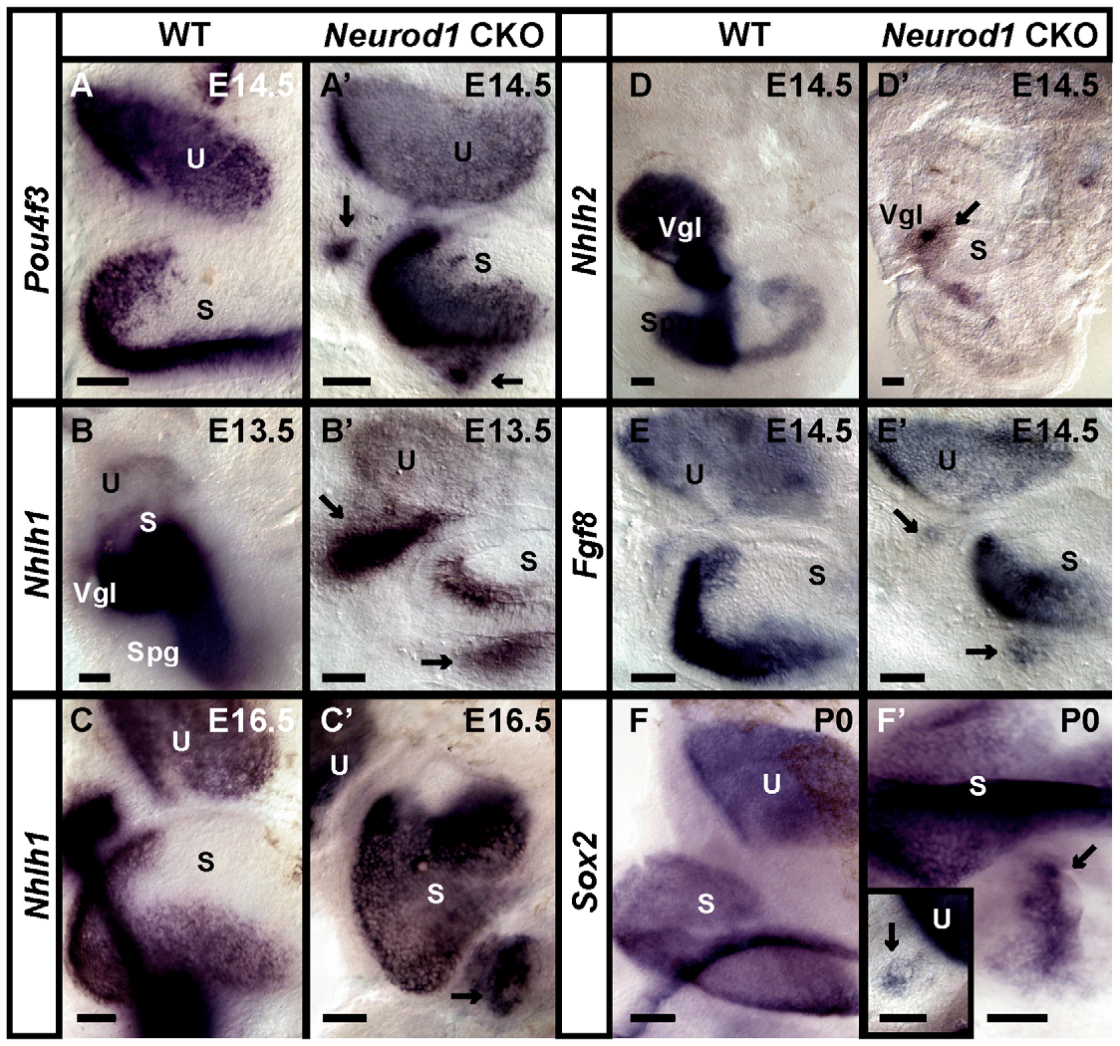
*Neurod1* suppresses hair cell specific genes in ganglia. While Myo VIIa and *Atoh1* are good indicators for the possible hair cell formation inside the ganglia of *Neurod1* CKO mice, we investigate the expression of other hair cell specific genes. *Pou4f3* and *Nhlh1* are expressed in hair cells (A–C) and responsible for their differentiation and are found in the ganglia in CKO mutants (A’–C’). In addition to hair cell marker, we also examine the neuronal marker *Nhlh2* (D,D’) and neurosensory marker *Fgf8* (E,E’) and *Sox2* (F,F’). We found only few *Nhlh2* positive neurons in the same topology near the intraganglionic vesicles in mutants suggesting a mix of hair cells and neurons near these vesicles (D’) as is clearly the case in histological sections (Fig. 1 K,K’, K”). *Fgf8* (E’) and *Sox2* (F’, insert in F’), which participate in neurosensory development, are also found near intraganglionic vesicles near the utricle and saccule. However, *Sox2* expression may also indicate formation of some supporting cells in those vesicles. (F’, insert in F’). S, saccule; U, utricle; Vgl, vestibular ganglia; Spg, spiral ganglia. Arrows indicate labeled cells inside the ganglia of *Neurod1* CKO mice. S, saccule; U, utricle; Vgl, vestibular ganglia; Spg, spiral ganglia. Bar indicates 100 µm.

While these data would normally be considered as proof of the hair cell nature of these cells [12], we worked with additional specific hair cells markers to establish that these cells were indeed hair cells and not transformed neurons with abnormal properties. Two POU domain factors are uniquely expressed in hair cells and neurons [33], [34], *Pou4f3* and *Pou4f1* (formerly *Brn3c* and *Brn3a*, respectively). We demonstrated that *Pou4f3,* an exclusive marker for hair cells in the ear with limited expression outside the ear [33], [35], was expressed in hair cell-like cells in the remaining ganglia of the *Neurod1* CKO ear (Fig. 3H) and colocalized with Myo VIIa (data not shown) identical to *Atoh1* (Fig. 2H, H’). The expression of these three markers (*Atoh1,* Myo VIIa and *Pou4f3)* is uniquely associated with hair cells in wild-type inner ears (Fig. 2C, 3). Therefore, their expression in cells within the remaining ganglia in *Neurod1* CKO mice provides evidence that these cells are hair cells. Differentiation of these hair cells started around the same time those markers were upregulated in the nearby vestibular sensory epithelia and, transiently, in the delaminating sensory neurons.

To further expand the notion that these cells were genuine hair cells, we next studied another set of bHLH genes *Nhlh1* and *Nhlh2* which are associated with hair cells and neurons, respectively [36]. *Nhlh1* and *Nhlh2* are primarily expressed in sensory neurons in early embryos with an additional later expression of *Nhlh1* in inner ear sensory epithelia [36]. We observed *Nhlh1* mRNA expression in the vestibular ganglia and in delaminating cells in early embryos with progressive upregulation in the sensory epithelia in later stages (Fig. 3B,C). In the absence of *Neurod1* the expression of *Nhlh1* was massively reduced in neurons with some residual expression in cells below the saccule and utricle (Fig. 3B’,C’). In contrast to *Nhlh1*, *Nhlh2* expression was exclusively in sensory neurons (Fig. 3D). In *Neurod1* CKO mice, *Nhlh2* expression was retained only in a small set of cells near the utricle (Fig. 3D’). Loss of expression of both genes in neurons was likely associated with early onset of apoptosis in *Neurod1* mutant [29].

We also investigated *Sox2*, a protein required for hair cell differentiation [37] and later is highly expressed in supporting cells in the ear [38]. Expression of this gene was also found within the ganglia next to the utricle and saccule (Fig. 3F’). This could indicate that some cells in these ganglia were possibly supporting cells.

**Figure 4:**
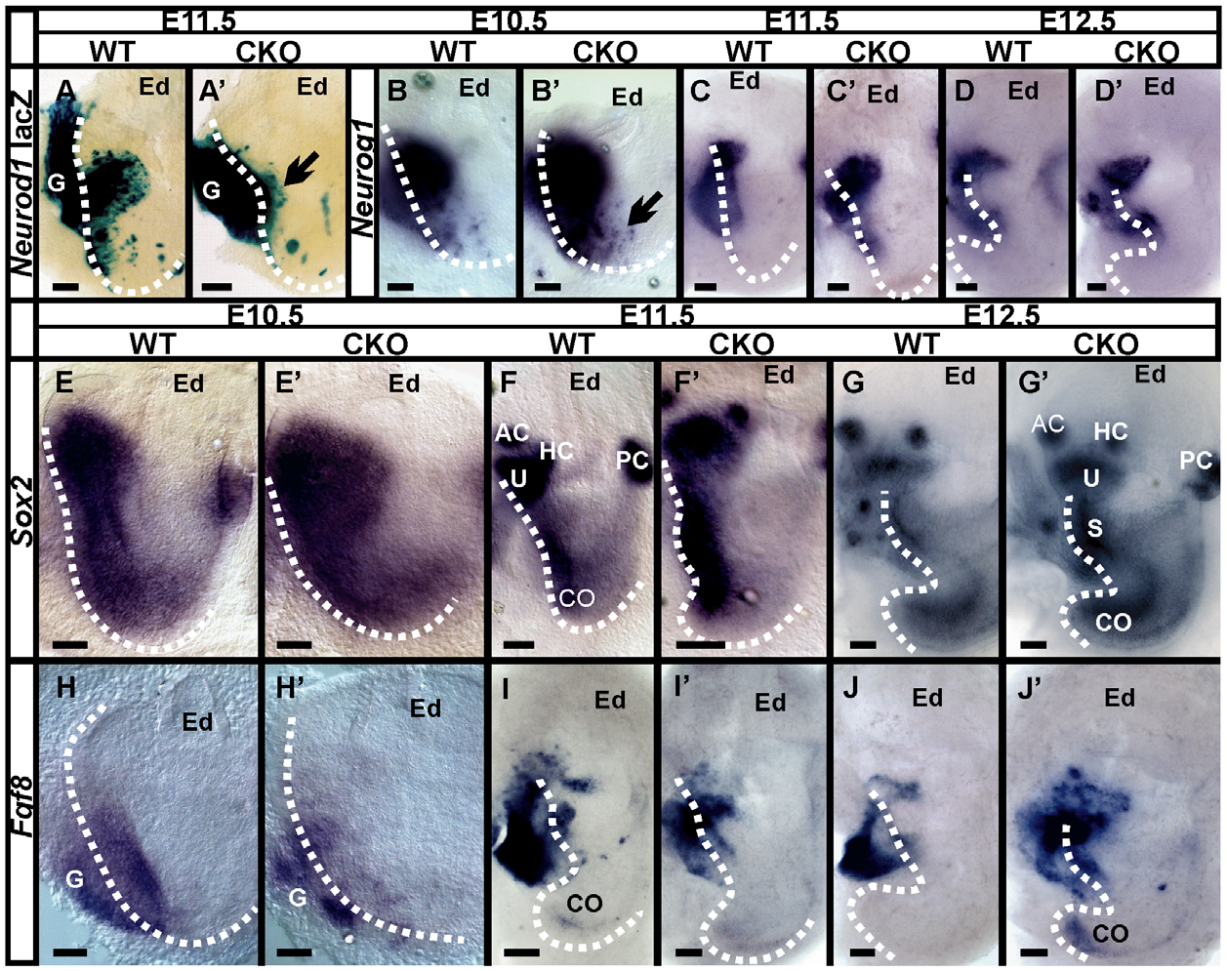
Absence of *Neurod1* results in aberrant expression of genes responsible for prosensory specification. At E11.5 embryo, β-galactosidase histochemistry show *Neurod1*-lacZ expression in the delaminating neuroblast (A, A’) and in the vestibular ganglion (G) which is moderately larger in the *Neurod1* CKO mice *(A’)*. In contrast, expression in the otic vesicle is reduced in *Neurod1* CKO mice *(arrow in A’). Neurog1,* an upstream regulator of *Neurod1*, show a larger expression in the absence of *Neurod1* (B’, C’, D’). In particular the prosensory domain is remarkably enlarged inside the otic vesicle with aberrant migration of the prosensory precursors (arrow in B’) in *Neurod1* CKO mice. After the specification of the sensory epithelia, *Neurog1* expression is progressively downregulated in wild-type mice (C, D) but some expression remains in the *Neurod1* CKO mutant (C’, D’). In the mutant, *Sox2* expression is expanded in the otocyst (compare E, E’) and shows more profound expression in the neurosensory precursor domain (F’,G’). We also investigate *Fgf8* expression which is strongly positive in the delaminating neuroblasts both in wild-type and in *Neurod1* CKO mice (H–J’). In the wild-type, *Fgf8* disappears in the otic vesicle after E11.5 but remains transiently restricted to the ganglia (I, J). In contrast, this *Fgf8* expression remains in the absence of *Neurod1* inside the otic vesicle in areas identified to be composed of sensory precursor cells (Fig. H’, I’, J’). G, vestibular ganglion; AC, anterior canal crista; HC, horizontal canal crista; PC, posterior canal crista; S, saccule; U, utricle;Co, cochlea; Ed, endolymphatic duct. Boundary of otic vesicle is marked with dotted lines. Bar indicates 100 µm.

Another factor exclusively expressed in some hair cells and only transiently in sensory neurons is *Fgf8* [39]. Consistent with data on several transcription factors, we found transient expression of *Fgf8* in delaminating sensory neurons of both wild-type and *Neurod1* CKO mice (Fig. 4H–J’). At later stages, when other markers for hair cells are expressed, we found *Fgf8* expression in the ganglia in a pattern reminiscent of the hair cells identified by other markers (Fig. 3 E’).

In summary, the presence of MyoVIIa, *Atoh1*, *Pou4f3*, *Sox2, Nhlh1*, *Nhlh2 and Fgf8* expression in cells near intraganglionic vesicles inside the remaining ganglia implied a substantial modification of the cellular identity of these cells in *Neurod1* CKO mice. We suggest that at least some surviving neurons are converted into hair cells which organize the surrounding tissue into vesicles inside ganglia and possibly regulate supporting cell differentiation of nearby neurons, fibroblasts or Schwann cells to form epithelia-like structures. Such organizing capacity of *Atoh1* expressing cells has already been demonstrated *in vitro* [40] and is known for *Fgf8 in vivo* [41]. Since none of these markers ever appear in neural crest derived Schwann cells or mesenchyme derived fibroblasts, it seems unlikely that they are *de novo* expressed in these cells in the absence of *Neurod1* expression in differentiating neurons. In contrast, several of these factors are known to be expressed in sensory neurons [17]. We therefore suggested that among the three cell types found in wild-type ear ganglia, it is the sensory neurons that are converted into hair cells. While highly suggestive of a neuronal origin, these data cannot fully exclude the alternative but more complex scenario of a Schwann cell or fibroblast transformation into hair cells in the absence of *Neurod1*.

### *Neurod1* affects hair cell type differentiation through regulation of multiple genes

A role of *Neurog1* in inner ear neurosensory lineage acquisition is well established along with *Neurod1* as a downstream activator of *Neurog1* [2], [15], [16]. We now provide evidence for a novel function of *Neurod1* consistent with its early expression in the prosensory domain to suppress an alternate hair cell fate. To further understand the possible interactions we analyzed in a *Neurod1* lacZ reporter the level of β-galactosidase expression in delaminating neuroblasts (Fig. 4A). We previously showed by *in situ* hybridization that *Pax2-Cre* results in complete and early deletion of *Neurod1* in mutant mice [29]. We therefore needed to use the reporter as both wild-type neurons and neurons lacking *Neurod1* will show the reporter. In contrast to wild-type mice, the expression of lacZ reporter was increased in the delaminating neurons of *Neurod1* CKO mice (Fig. 4A’) while the expression in the otocyst was reduced. This early knock out of *Neurod1* resulted in considerable changes in expression pattern of several genes analyzed in different embryonic stages with *in situ* hybridization (Fig. 4).

For example, *Neurog1* persisted longer in its expression in the delaminating neurons in the absence of *Neurod1* (Fig. 4B’,C’,D’), suggesting a negative regulation of *Neurog1* by *Neurod1*. *Sox2* is associated with progenitor and stem cell populations in the developing CNS tissues [42], which might also be true for the sensory progenitors of the cochlea [37], [43]. We therefore also investigated *Sox2* expression in early embryos of *Neurod1* mutant mice. In *Neurod1* CKO mice, *Sox2* expression was expanded most prominently in the dorsal vestibular but also in the ventral prosensory region of the cochlea (Fig. 4E’,F’,G’) compared to the wild-type littermates (Fig. 4E, F, G). Later expression of *Sox2* was near the ectopic vesicles inside the ganglia (Fig. 3F’).

Among twenty-three different Fgfs and four FGF receptors, several are known to function in early otic induction and sensory specification [8], [44], [45], [46], [47]. We investigated *Fgf8* expression due to its known early expression in delaminating neurons and later function in organ of Corti in supporting cell development [41], [48], [49]. We found *Fgf8* expression in the early stage of development, which is substantially modified in the absence of *Neurod1* (Fig. 4H–J’). *Fgf8* was expressed in the prosensory domain and transiently in the delaminating neuroblasts (Fig. 4H,I,J) of wild-type mice. In *Neurod1* CKO mice, *Fgf8* was expressed in delaminating cells with moderate loss of expression in ganglia (Fig. 4H’,I’,J’) consistent with the neuronal loss in the absence of *Neurod1* [29].

In summary, we suggested a compelling role of *Neurod1* in specification of neurosensory precursors beyond its role in neuronal differentiation, possibly through an interaction with important genes like *Neurog1, Sox2 and Fgf8*. This suggestion is based on the fact that early deletion of *Neurod1* results in substantial changes of expression of several genes that may directly or indirectly affect cell fate determination of the precursor population.

### Inactivation of *Neurod1* results in a shortened and disorganized organ of Corti

Having now established that *Neurod1* affects cell fate acquisition in the ear ganglia we investigated the effect of loss of *Neurod1* on hair cells. Previous work has shown a surprising overlap of apparent cell specific bHLH genes in inner ear development. For example, *Neurog1* null mice not only lose all sensory neurons but also have truncated development of hair cells in several sensory epithelia [14], [17]. Recently, a lineage relationship between neurons and some hair cells was demonstrated [20]. Interestingly, despite absence of *Neurog1*, hair cells strongly express *Neurod1* [17], suggesting that hair cell specific factors such as *Atoh1* are also able to upregulate *Neurod1* expression and can do so more effectively in the absence of *Neurog1*. Since *Neurod1* is immediately downstream and directly regulated by *Neurog1* [2], we investigated the effects of *Neurod1* on hair cell development as they are affected by the absence of either *Neurog1* or *Neurod1* [14], [15], [16], [20]. Consistent with an expression of *Neurod1* in hair cells [29] and previous suggestions about a shortened cochlea with disorganized hair cells in *Neurod1* systemic null mice [15], [16], [50], we also found a truncation of the cochlea. When compared to wild-type, *Neurod1* mutant cochlea was not as much shortened as *Neurog1* null cochlea (Table 1). Size reduction in *Neurod1* CKO mice was also apparent in other epithelia and canal cristae which were approximately 30% shorter than the control (Table 1; Fig. S1). These data suggest that *Neurod1* exerts a comparable effect on hair cell formation as *Neurog1* albeit at a reduced scale. The somewhat less severe effect could relate to the fact that *Neurod1* is downstream to *Neurog1* and some common sensory neuron/hair cell precursors may already have separated.

**Table 1:** Length of the Cochlea in wild-type, *Neurod1* CKO and *Neurog1* null mutations.

Length of Cochlea (µm)	WT (n = 4)	Neurod1 CKO (n = 4)	Neurog1 null (n = 3)
Mean	5907	2695	2449
SD	408	138	320

**Figure 5:**
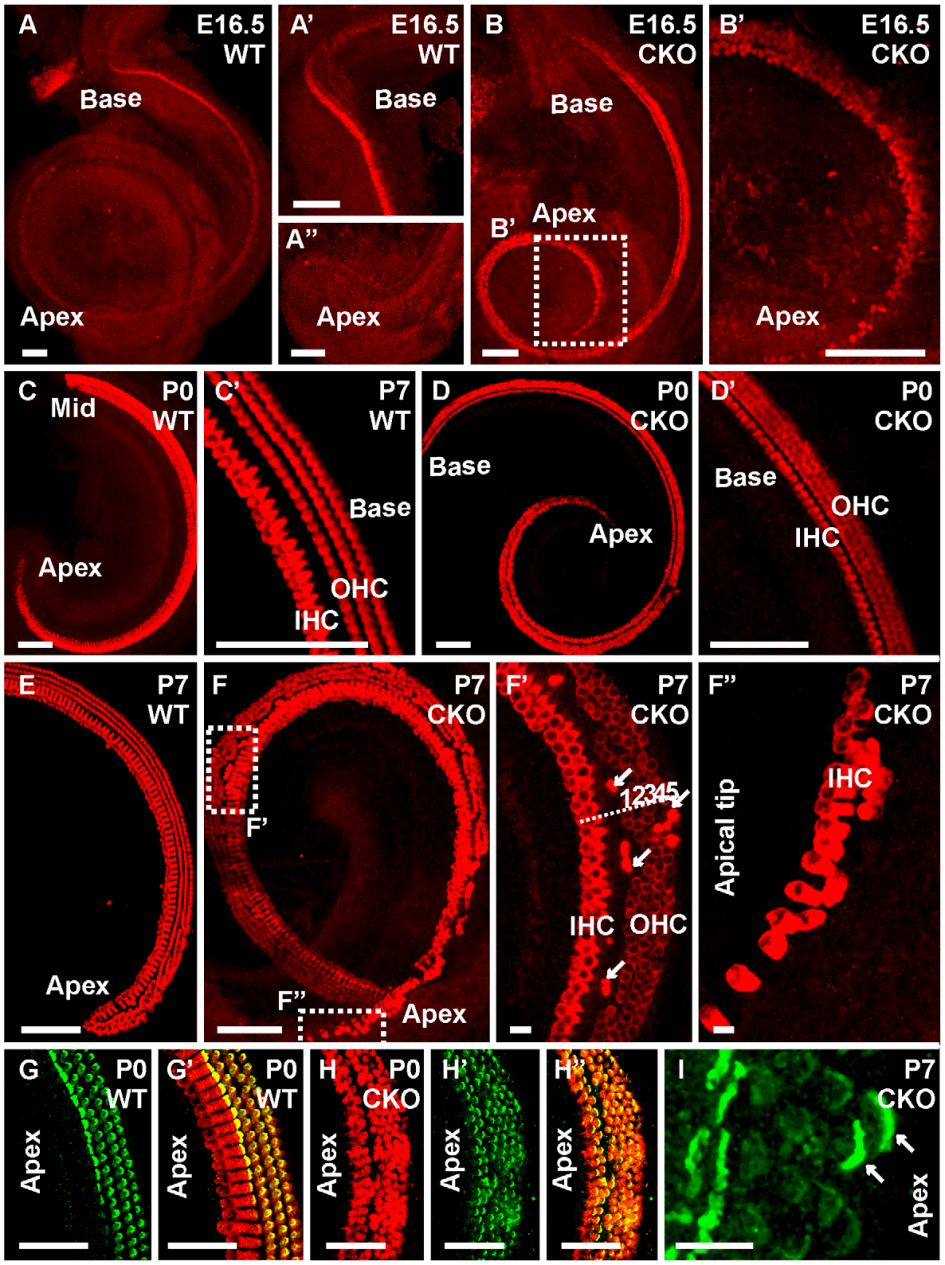
*Neurod1* is necessary for development of an orderly patterned organ of Corti. Myo VIIa immunocytochemistry shows upregulation of Myo VIIa in the wild-type starts around E16.5 from the mid-base and later progresses toward both base and apex with a regular organization of one row of inner and three rows of outer hair cells throughout the cochlea (A, A’, C, C’, E). In contrast, Myo VIIa is already expressed throughout the cochlea with disorganization of hair cells in the apical half (B, B’, D) in E16.5 *Neurod1* CKO mutant littermates. In later stages, the basal half of the CKO mutant shows normal orientation of the hair cells (D, D’) whereas the apex of *Neurod1* CKO mice shows multiple rows of both IHCs and OHCs with reduction of Myo VIIa intensity in most outer hair cells (F, F’). In addition, clusters of higher intensity of Myo VIIa positive cells are found in between outer hair cells with equivalent staining intensity to inner hair cells (arrows in F’). The apical tip of the mutant cochlea shows a partially duplicated row of inner hair cells with complete absence of outer hair cells (F”). Using espin immunocytochemistry we confirm the disorganization of the apical half of the mutant cochlea where two rows of inner hair stereocilia and four to five rows of outer hair stereocilia are observed (H–H’) along with some unusually displaced strongly stained inner hair stereocilia (arrow in I) in between faintly labeled outer hair stereocilia (I). IHC, inner hair cells; OHC, outer hair cells. Bar indicates 100 µm except F”; 10 µm in F”.

We next wanted to investigate whether the truncated cochlea has multiple rows of disorganized hair cells as previously reported in other mutants with shortened cochlear growth [9], [14]. There was a gradient of malformation of the hair cells in the base and apex of *Neurod1* CKO mice (Fig. 5) as demonstrated with Myo VIIa immunofluorescence staining at different stages of development. We observed premature expression of Myo VIIa in the apex of *Neurod1* mutant cochlea in comparison to wild-type mice (Fig. 5A,A”,B,B’). In contrast, the organization of the organ of Corti was normal in the basal half of the cochlea and comparable with the control mice (Fig. 5C–D’). However, the orientation of hair cells was severely disrupted in the apical half. In this location, multiple rows of inner and outer hair cells (IHCs, OHCs) were found (Fig. 5F). The misalignment of the hair cells in the apical half of mutant cochlea was more obvious in later stages (P7 shown here) and demonstrated two rows of IHCs and four to five rows of OHCs (Fig. 5F,F’). In addition, Myo VIIa was more prominently expressed in IHCs in the apex, as compared to the uniform expression in the base, and similar high levels of expression of Myo VIIa was found in scattered OHCs in the apex (Fig. 5F,F’). We therefore interpreted these cells as ‘ectopic IHC’s’. The apical tip consisted exclusively of IHCs in two disorganized rows without any OHCs (Fig. 5F”).

To evaluate the apical specialization of the organ of Corti of *Neurod1* null mice, we performed the espin immunofluoresence labeling combined with the Myo VIIa. We found similar abnormality of stereocilia with espin as with Myo VIIa in hair cells in the apical half. The espin immunolabeling confirmed the disorganization of organ of Corti in the apex of *Neurod1* CKO mice where multiple rows of IHCs and OHCs were found with two distinct types of stereocilia (Fig. 5H–H”). In the newborn mice, density of the stereocilia of both IHCs and OHCs were almost equal, but at later stages the density of stereocilia of OHCs was remarkably reduced with few displaced highly dense inner hair stereocilia in place of weakly labeled outer hair stereocilia (Fig. 5I). Near the apical tip only the inner type of stereocilia were found (data not shown).

**Figure 6:**
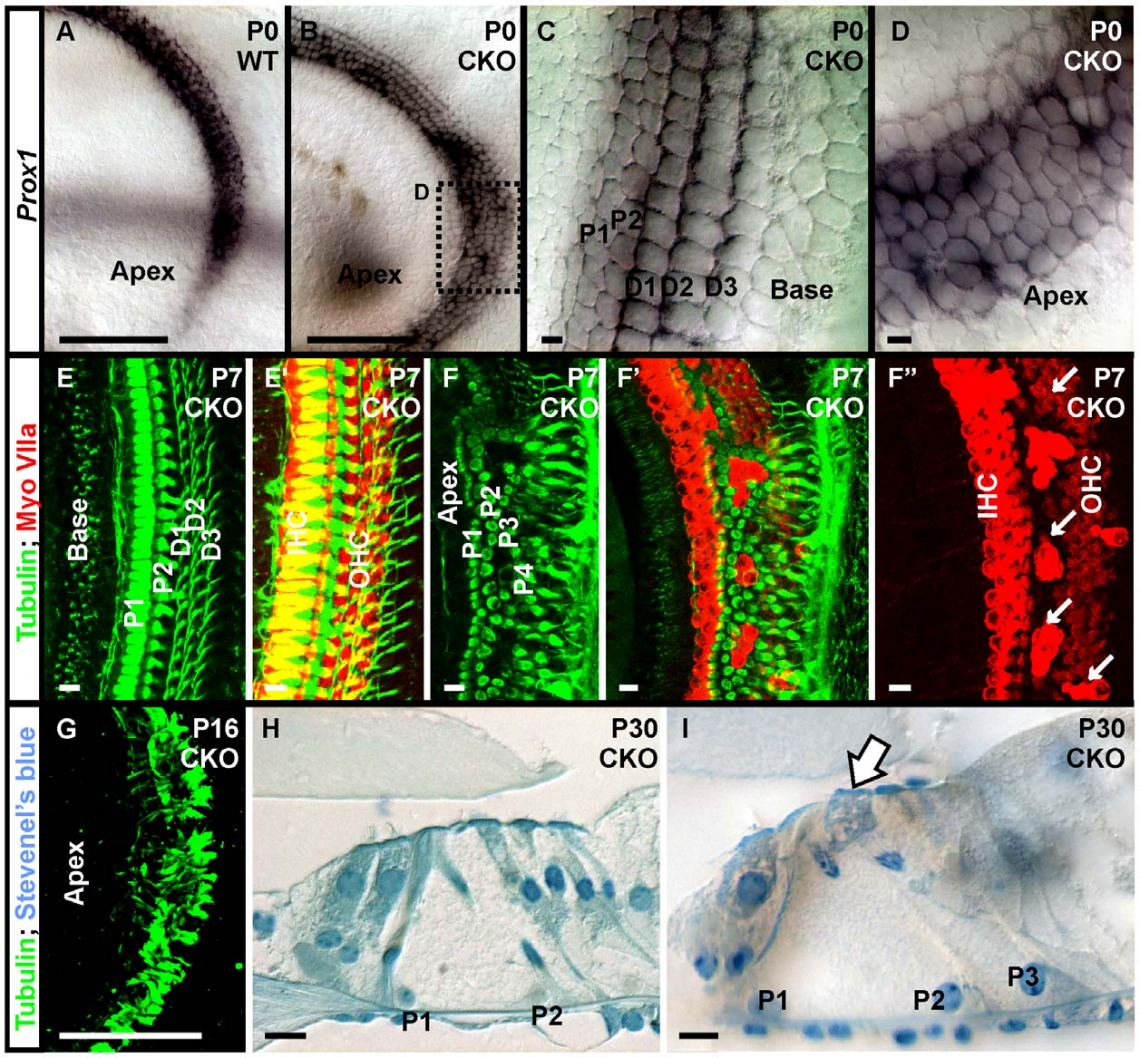
*Neurod1* regulates organization of supporting cells as well as hair cells. A supporting cell marker, *Prox1*, shows uniform expression in two rows of pillar and three rows of Deiter’s cells in wild-type mice (A) and in the base of *Neurod1* CKO mice (B,C). In contrast, the apex of *Neurod1* CKO mice shows multiple disorganized rows of supporting cells with no clear distinction between Pillar and Deiter’s cells (D). At P7, combined tubulin and Myo VIIa immunocytochemistry shows normal organization of supporting cells in between hair cells in the base of the mutant cochlea (E, E’). This orientation is disrupted in the apex where Myo VIIa positive hair cells are surrounded by supporting cells with thick processes filled with tubulin. This is reminiscent of Pillar cells and not of the thinner phalangeal processes of Deiter’s cells (F, F”, G). Detailed histology in thin plastic sections of P30 mutant cochlea show not only the persistence of multiple rows of inner and outer hair cells in the apex (H) but also the formation of multiple rows of Pillar cells (P1, P2, P3) and inner hair cells in places of outer hair cells (arrow in I). These data suggest that *Neurod1* is an important transcription factor that mediates the coordinated type specific hair cell and supporting cell development in the apical half of the organ of Corti which is necessary for the coordinated development of supporting cells. P1, P2, inner and outer Pillar cells; D1, D2, D3, 1^st^, 2^nd^, 3^rd^ row of Deiter’s cells; IHC, inner hair cells; OHC, outer hair cells. Bar indicates 100 µm except C, D, H, I and 10 µm in C, D, H, I.

We next wanted to understand how the irregularity of hair cells affected the supporting cells. *In situ* hybridization with *Prox1*, a marker for Deiter's and Pillar cells [51], [52] revealed disorganized supporting cells (Fig. 6D). We also used β-tubulin immunofluoresecnce labeling in P7 and P16 *Neurod1* CKO mutant mice (Fig. 6E–F’,G). We found the organization of supporting cells in the basal half of the cochlea was regular with thick processes of two Pillar cells and thin phalangeal processes of Deiter’s cells labeled with β-tubulin (Fig. 6E, E’). In the apical half, most Deiter’s cell processes were converted into thick Pillar cell processes (Fig. 6G) that surrounded the ectopic, strong Myo VIIa positive IHCs (Fig. 6F–F”).

We further investigated this unusual phenotype in later stage of serially sectioned P30 cochlea. At this stage, while some normal OHCs near the apex had developed a cigar shape pattern, some abnormal OHCs resembled IHCs in their shape (Fig. 6H,I) as well as duplication of IHCs consistent with the finding with Myo VIIa staining (Fig. 6H). In addition, the supporting cells around these hair cells were markedly disorganized and had multiple rows of Pillar cells (Fig. 6I).

### Apical disorganization may relate to premature expression of hair cell genes

**Figure 7:**
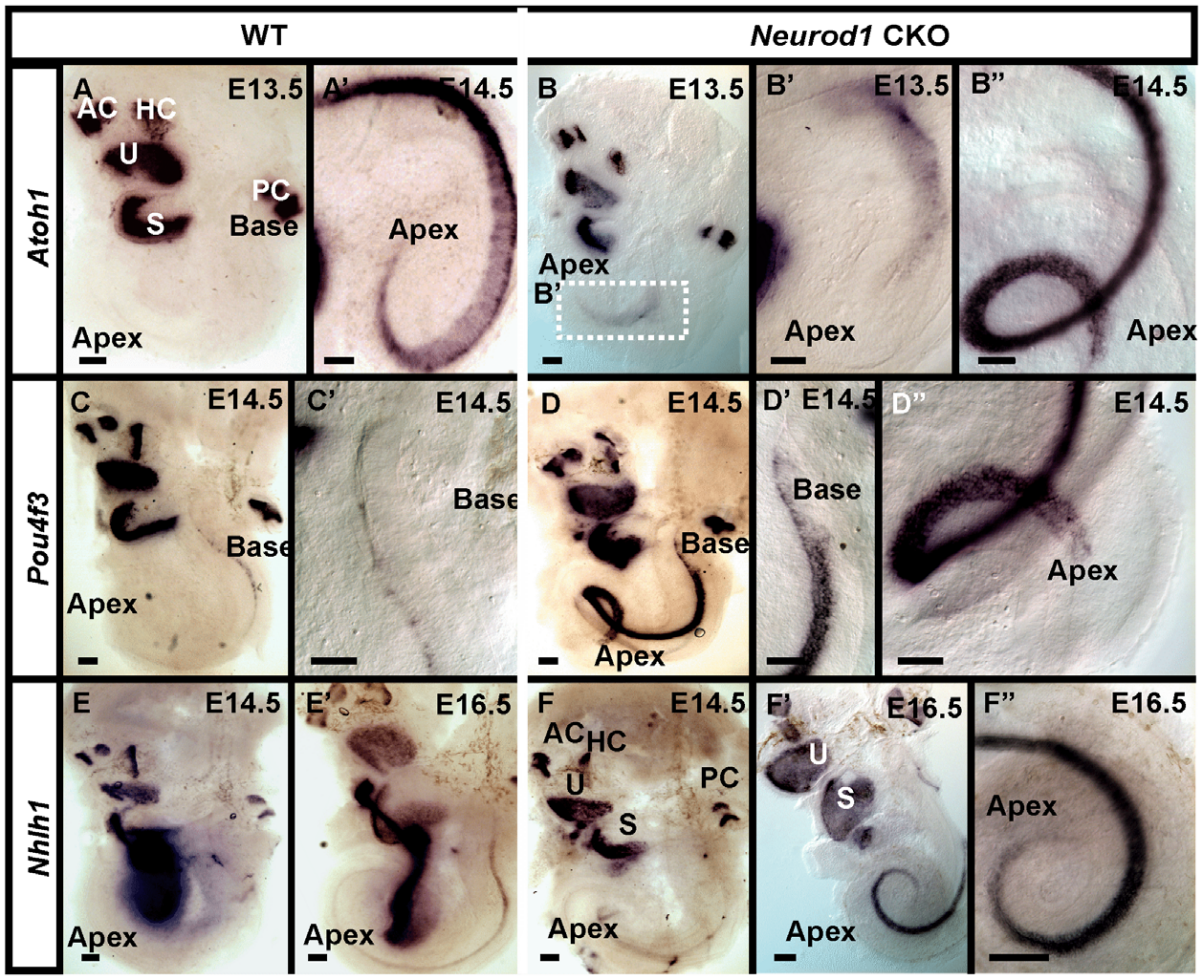
*Neurod1* controls hair cell specific gene expression in the apex. Normally hair cell cycle exit and differentiation is delayed in the cochlea compared to vestibular epithelia and shows a pattern of apex-to-base progression of cell cycle exit. In contrast, there is a base-to-apex progression of differentiation, including upregulation of *Atoh1* (A), *Pou4f3* (C) and *Nhlh1* (E) in wild-type mice. In *Neurod1* CKO mice, the expression of these genes not only happens earlier but progresses from the apex to the base (compare A and B; C and D, D”; E, E’ and F, F’,F”). *Atoh1* and *Pou4f3* are essential for hair cell differentiation and maintenance, respectively. Their expression suggests a premature initiation of hair cell differentiation that normally is delayed in the apex compared to the base. Note that the apex in B is shown with 90° anti-clockwise rotation in B’. AC, anterior canal crista; HC, horizontal canal crista; PC, posterior canal crista; S, saccule; U, utricle. Bar indicates 100 µm.

We next investigated several genes relevant for neurosensory ear development to elucidate further the extent of defect at the level of gene expression. *Atoh1* is a crucial factor for hair cell differentiation of the inner ear [1]. In the cochlea, *Atoh1* is upregulated in a base to apex gradient, starting around E13.5 [17], [32]. We found a spatiotemporally altered *Atoh1* expression in the *Neurod1* mutant cochlea (Fig. 7B–B”). *Atoh1* was expressed in the apex of *Neurod1* mutant cochlea (Fig. 7B–B’) as early as E13.5 before it appeared in the wild-type littermate cochlea (Fig. 7A). At E14.5, *Atoh1* was profoundly expressed in the entire cochlea of *Neurod1* mutant mice (Fig. 7B’) whereas in the wild-type littermate, expression was weak in the apex with an obvious gradient suggesting a base to apex progression (Fig. 7A’). Therefore, *Atoh1* expression gradient in the cochlea was not only altered in the absence of *Neurod1* but also was expressed earlier than in the wild-type mice (compare Fig. 7A, B). A similar phenotype with premature expression of *Atoh1* in the apex was reported in *Neurog1* null mice [17]. This similarity could indicate a possibly similar mechanism as *Neurod1* is downstream of *Neurog1* [2] and that *Neurog1* could directly or indirectly regulate *Neurod1* expression in the apical hair cells.

*Pou4f3* is responsible for cochlear and vestibular hair cell differentiation and survival. Deletion results not only in hair cell loss but also in delayed loss of ganglion neurons [33], [35], [53]. We have analyzed the expression of *Pou4f3* in *Neurod1* CKO mice with *in situ* hybridization. *Pou4f3* expression starts shortly after the hair cell fate is committed [54]. We found a premature upregulation of *Pou4f3* in the apex of the mutant cochlea much earlier than wild-type shown at E14.5 (Fig. 7D, D”) while in the wild-type littermate, *Pou4f3* expression had just started near the base of the cochlea (Fig. 7C, C’).

*Nhlh1* is later in development expressed in the hair cells of all sensory epithelia [36]. In wild-type mice, *Nhlh1* was expressed and upregulated in the base of the cochlea at E16.5 with an apparent base to apex gradient (Fig. 7E’). In *Neurod1* CKO mice, *Nhlh1* expression was already evident in the apex of the cochlea at E14.5 (Fig. 7F). By E16.5, *Nhlh1* was expressed in the entire cochlea of *Neurod1* CKO mice, which is obviously more profound in comparison to weak expression near the base of the wild-type littermate (compare Fig. 7E’, F’, F”).

In summary, Myo VIIa, *Atoh1*, *Pou4f3* and *Nhlh1* showed an altered pattern of expression in *Neurod1* CKO cochlea with premature expression in an apex-to-base instead of a base-to-apex progression as in wild-type littermates (Fig. 5A–B’, 7). This spatiotemporal aberration of the bHLH gene expression might alter the onset of differentiation of apical hair cells. The premature expression of these transcription factors in hair cells that show a delayed differentiation compared to their early cell cycle exit [17] may relate to the disorganization of apical hair cells in *Neurod1* CKO mice.

*Atoh1* expression was not only altered spatiotemporally but also persisted longer in the absence of *Neurod1*. For example, *Atoh1* expression progressively reduced in the wild-type cochlea from base-to-apex started from P0 to onward with restricted expression only in the inner two rows of OHCs (Fig. S1A–A’; shown in P0). In contrast, *Atoh1* expression persisted longer in all the hair cells throughout the cochlea of *Neurod1* CKO mice (Fig. S1B–B”). *Atoh1* was also aberrantly expressed in the non-sensory compartment which was most obvious in the cruciate eminence of the anterior cristae (AC) and also in striola region of the saccule and utricle (Fig. S1D–D”). Consistent with *Atoh1* expression, we also found longer persisting *Pou4f3* expression in the base and apex of the *Neurod1* CKO cochlea in contrast to wild-type littermates (data not shown). We previously reported that loss of *Neurod1* resulted in continued expression of *Atoh1* in cerebellar granule cells [26]. Apparently, *Neurod1* exerts a similar inhibitory influence on the expression of *Atoh1* and its downstream genes *Nhlh1* and *Pou4f3*.

### Altered *Fgf8* expression may relate to the cochlear histological changes

**Figure 8:**
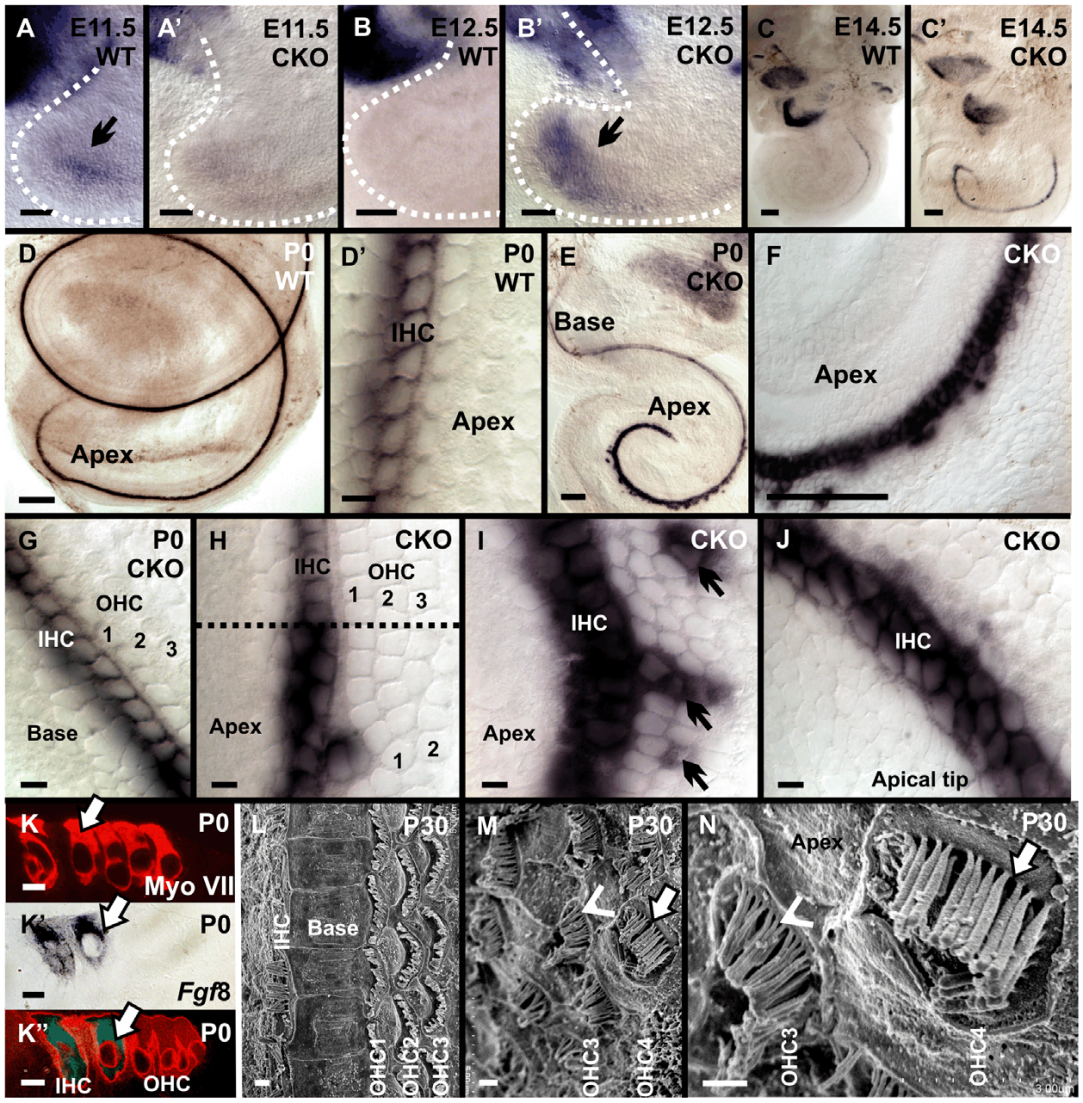
*Fgf8* misexpression correlates with formation of ‘ectopic inner hair cells’. In situ hybridization shows a persistent expression of *Fgf8* in neurosensory precursors in *Neurod1* mutant cochlea as early as E12.5 (arrow in B’) with premature expression in particular in the apex of the cochlea (C’) in comparison to wild-type (C). *Fgf8* is expressed transiently in the prosensory domain in wild-type mice (arrow in A) and later is shown to be upregulated in the cochlea with a base-to-apex gradient (A,B,C). In newborn mice, *Fgf8* is uniformly expressed in all inner hair cells almost along the entire length of the cochlea in wild-type mice (D, D’). In contrast, *Neurod1* CKO mice display an increased expression level in the apex (E, F, H, I, J) and deviate from the single row labeling of only inner hair cells seen in the base (G). Two or more rows of inner hair cells are positive for *Fgf8* and scattered single and multiple cells are interspersed among the multiple rows of outer hair cells which are also positive for *Fgf8* (arrows in I). The apical tip shows up to three rows of *Fgf8* positive cells (J). Radial sections through the apex of *Fgf8* ISH reacted and Myo VIIa immunostained cochlea reveals co-localization of *Fgf*8 and Myo VIIa in inner as well as ‘ectopic inner hair cells’ scattered among outer hair cells (arrows in K–K”). SEM of P30 *Neurod1* CKO mice reveals a normal organization of inner hair cells in a single row and three rows of outer hair cells in the base of Neurod1 CKO mice (L). In contrast, the apex shows inner hair cell sized stereocilia (arrows) interspersed among normal sized stereocilia bearing outer hair cells (arrowhead; M,N). Dotted line in H indicates border between normal and disorganized organ of Corti. IHC, inner hair cells; OHC, outer hair cells. Bar indicates 100 µm in A–F except D’; 10 µm in D’,G–J, K–K” and 1 µm in L–N.

In mice, FGF3, FGF8 and FGF10 play a role in the early inductive events of the otic vesicle formation [8], [39], [46], [55], [56], [57], [58]. We observed *Fgf8* expression in the delaminating sensory neuron in both wild-type and *Neurod1* CKO mice as early as E10.5 (Fig. 4). Consistent with expression changes in *Atoh1* and other downstream hair cell specific genes, *Fgf8* was also expressed prematurely in the apex of the *Neurod1* mutant mice (Fig. 8B’). *Fgf8* was transiently expressed in the apex of wild-type mice but disappeared after E11.5 (compare Fig. 8A and B). However, absence of *Neurod1* resulted in continued *Fgf8* expression in the apex from E11.5 onward and thus resulted in premature and reversed expression pattern (Fig. 8A’, B’, C’). In contrast, in wild-type mice, *Fgf8* expression started at E14.5 from the base of the cochlea progressing over time to the apex (Fig. 8C).

In the cochlea, Fgf8 is expressed in IHCs from where it diffuses to bind to its receptor, Fgfr3, which leads to the development of Pillar cells instead of Deiter’s cells [41], [59], [60]. Our data on Myo VIIa expression in the *Neurod1* CKO mutant suggested that some OHCs may achieve an inner hair cell-like phenotype (‘ectopic IHCs’; Fig. 5) and this may be due to an altered *Fgf8* expression. We therefore examined *Fgf8* expression to verify that these cells are ‘ectopic IHCs’ as Fgf8 is a marker of IHCs of the organ of Corti [44]. In wild-type mice, *Fgf8* was expressed exclusively in the single row of IHCs (Fig. 8D,D’). In the *Neurod1* CKO mutant, we found single rows of *Fgf8* positive IHCs only in the basal half of the cochlea (Fig. 8E,G) whereas in the apical half, multiple rows of *Fgf8* positive IHCs was observed as well as ectopic expression in some IHCs replacing OHCs (Fig. 8E,F,H,I) consistent with the observation of Myo VIIa expression (Fig. 5F,F’). Closer to the apical tip we found only *Fgf8* expression in multiple rows of IHCs without OHCs (Fig. 8J). In conclusion, absence of *Neurod1* altered *Fgf8* expression in the apex of the mutant cochlea which directly or indirectly related to the change of stereotyped pattern and differentiation of apical hair cells in the organ of Corti.

To analyze more closely the effect of *Fgf8* expression in the organ of Corti, we performed plastic sections of *Fgf8 in situ* reacted P0 ears sequentially immunolabeled with anti Myo VIIa antibody. Sections through the base showed the expected distribution of a single row of *Fgf8* and Myo VIIa positive IHCs and three rows of OHCs (data not shown). While,radial sections through the apex confirmed both clusters of inner hair cells as well as single or multiple *Fgf8* positive cells among the OHCs in *Neurod1* CKO mice (Fig. 8K–K”) consistent with the whole mounted data.

We further investigated the consequences of *Fgf8* misexpression and analyzed the differentiation of the stereocilia in P30 mice using SEM. Consistent with the patchy expression of *Fgf8* in only some topographic OHCs we found a patchy aberration of stereocilia where some ‘OHCs’ had stereocilia twice as thick as others, resembling the diameter of inner hair cell stereocilia (Fig. 8M,N). The changes in stereocilia supported the evidence of ‘ectopic IHCs’, dispersed among OHCs.

In summary, absence of *Neurod1* leads to premature upregulation of hair cell differentiation genes in the apex, severe disorganization of the apical hair cells and supporting cells, misexpression of *Fgf8* in some ‘OHCs’, and development of inner hair cell-like stereocilia among OHCs. Consistent with its early expression in the inner ear prosensory region, *Neurod1* plays a significant role in hair cell maturation through the suppression of several genes in the apex, most prominently *Atoh1* and *Fgf8*. We tested whether a delayed knockout of *Neurod1* using *Tg(Atoh1-cre)* could achieve these altered differentiation of hair cells. Despite a massive cerebellar phenotype of this CKO mouse [26], our data showed no effect in inner ear development or any alteration of phenotype of neurosensory cells (Fig. S2). Once the prosensory domain is specified in early embryonic stage, later deletion of *Neurod1* has no effect in refinement of hair cell fate.

## Discussion

*Neurod1* is essential for neuronal differentiation in the cerebellum [26] and the ear [29] and can convert non-neuronal cells into neurons [61] through the regulation of over 500 downstream genes [62]. We analyzed the role of *Neurod1* in inner ear neurosensory cell development using a newly generated *Neurod1* conditional knockout mouse. We previously reported [15], [29] substantial loss of inner ear sensory neurons and disorganization of remaining afferent projections in *Neurod1 systemic and conditional null* mice. The expression of other bHLH genes such as *Nhlh1* and *Nhlh2* [36] may be responsible for partial rescue of those few sensory neurons that survive in the absence of *Neurod1*. We here identify two novel roles of *Neurod1*:

1. *Neurod1* suppresses hair cell differentiation in sensory ganglia.
2. *Neurod1* controls gene expression needed for outer hair cell maturation.

### *Neurod1* suppresses differentiation of ganglion cells into hair cells

A cascade of pro-neuronal bHLH genes transforms ectodermal cells into neurons and can do so by simply being misexpressed in the developing ectoderm [61] or the ear [43]. These bHLH proteins also determine cell fate in other tissues such as pancreas [16], gut [63] and Merkel cells [64]. bHLH genes are important for cell fate switch. Without expression of *Atoh1,* cells can change from a secretory to an absorptive phenotype [65].

In this study, we observed the formation of vesicles lined by hair cells in place of remaining ganglia in the *Neurod1* CKO mice (Fig. 1). To further understand the molecular basis of formation of these ‘intraganglionic hair cells’, we analyzed expression of multiple hair cell specific genes. We found positive expression of *Atoh1* and *Pou4f3* and the hair cell marker Myo VIIa in these ‘intraganglionic hair cells’. *Nhlh1* and *Neurod1* are limited early on to neuronal expression but are found later in hair cells, including the ‘intraganglionic hair cells’ (shown with *Nhlh1 in situ* hybridization and *Neurod1* lacZ expression). The overlapping function of *Neurod1* and *Nhlh1* may not only lead to the survival of some neurons but the absence of *Neurod1* may allow premature and persistent expression of *Atoh1* in these remaining ganglion neurons (Fig. 2D, D’). Consistent with the previous report of *Atoh1* expression in some neurons [17], our *in situ* data also show a transient *Atoh1* expression in the delaminating sensory neurons in the wild-type embryo. This limited expression of *Atoh1* may normally be restrained by the expression of *Neurod1* and absence of *Neurod1* seems to allow continued expression. We suggest that this continued expression of *Atoh1*, combined with the absence of *Neurod1* expression can result in the differentiation of ‘intraganglionic hair cells’ as well as the expression of other hair cell markers such as Myo VIIa, *Pou4f3* and *Nhlh1*. In addition to hair cell markers, these or other cells within the ganglia show neurosensory markers such as Sox2, *Nhlh2* and *Fgf8*.

Only three cell types with different embryonic origins are found in vestibular and cochlear ganglia: inner ear derived sensory neurons [66], mesoderm derived fibroblasts and neural crest derived Schwann cells [67]. A transformation of Schwann cells or fibroblasts, which never express *Neurod1*, is theoretically possible. However, the presence of multiple genes known to be expressed in neurosensory cells but not in Schwann cells or fibroblasts makes this a very improbable scenario. We therefore interpret our data to suggest that some inner ear derived ‘sensory neuron precursors’ adopt a hair cell fate in the absence of *Neurod1*. Such ‘hair cell’ formation from delaminated neurosensory precursors suggests a degree of flexibility of cell fate acquisition and is consistent with the emerging concept of lineage and possibly clonal neurosensory relationships in the ear [18], [19], [20]. In addition, these Myo VIIa positive ‘hair cells’ express marker genes otherwise only associated with hair cells in the ear, and reside around vesicles inside vestibular ganglion aggregations near the utricle and saccule. Since none of these markers ever appear in neural crest derived Schwann cells or in fibroblasts but at least *Atoh1* is known to be expressed in sensory neurons [17], we suggest that of all three cell types found in wild-type ear ganglia it is the sensory neurons that are converted to hair cells. We name these hair cells as ‘intraganglionic hair cells’. Further work is needed to analyze in details the transformation of sensory neuron precursors into ‘intraganglionic hair cells’ and demonstrate the suppression of *Atoh1* by *Neurod1* at the molecular level either within a given cell or between cells via the delta/notch system.

### *Neurod1* helps to organize the organ of Corti by controlling spatiotemporal gene expression

Previous work showed that *Neurod1* is expressed in hair cells [16], [17] in a shorter and disorganized cochlea [15], [50] of *Neurod1* null mice. It has also been noted that some of the first row of OHCs obtain inner hair cell like appearance [16] and the IHCs may form multiple disorganized rows. We demonstrate for the first time the degree of disorganization of the cochlear apex with the formation of ectopic IHCs in place of OHCs in *Neurod1* CKO mice and show the complete absence of OHC formation in the most apical part of the cochlea. We show expression of IHC marker genes in cells that topologically should be outer hair cells and the histological alteration of these cells such as diameter of stereocilia. *Neurod1* mimics *Neurog1* with respect to shortening, disorganization and gene expression alteration in the cochlea [14]. Like in *Neurog1* null mice, *Atoh1* is prematurely upregulated in the apex of *Neurod1* CKO mice, suggesting a fate change of common precursors toward hair cells [17]. The most reduced sensory epithelia in *Neurog1* null mice is the saccule [14], [31], now known to be affected because of lineage relationship of saccular neurons and hair cells [20].

In contrast, *Neurod1* CKO mice show the most profound size reduction in canal cristae and cochlea (Table 1, Fig. S1B,D,D”). Effects of *Neurod1* on overall growth are thus not simply a milder extension of *Neurog1* effects. It is possible that simple premature expression of *Atoh1* and its downstream genes disrupts convergent extension [68] and thus leads to the observed histological alteration of the cochlear apex. This is in agreement with the complete extension of the cochlea in the absence of *Atoh1* expression and any differentiation of hair cells [31]. Other mutants with reduction in growth and multiple rows of hair cells show no mixing of inner and outer hair cells [9], [14]. Combined with the enhanced cell death as early as E9.5 in *Neurod1* null mice [29], the early appearance of truncated growth in the cochlea suggests that common neuronal/hair cell precursors may die in the absence of *Neurod1*, reducing the growth of the organ of Corti and canal cristae. The expression of other bHLH genes such as *Nhlh1* and *Nhlh2* may rescue some common neurosensory precursors in the utricle and saccule resulting in near normal size growth. What additional gene(s) may mediate these differential sensory epithelia effects is unknown.

### How can *Neurod1* affect neuronal and hair cell differentiation?

**Figure 9:**
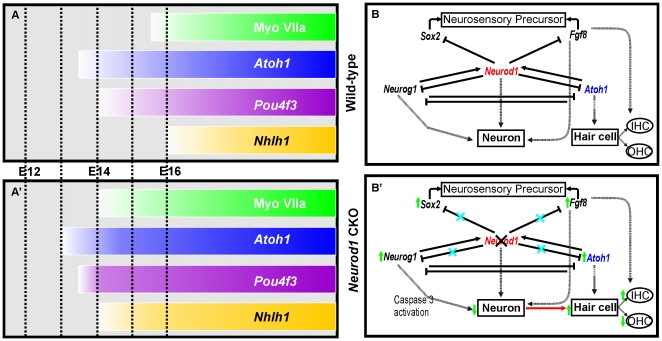
*Neurod1* regulates neuronal differentiation by suppression of premature hair cell differentiation of neurons possibly interacting with several target genes. In the absence of *Neurod1* several hair cell specific genes such as Myo VIIa, *Atoh1*, *Pou4f3* and *Nhlh1* are prematurely expressed with an inverse gradient of apex-to-base progression of hair cell differentiation instead of usual base-to-apex progression (cochlear expression shown with bars in A, A’). In addition, these genes are also expressed ectopically in the differentiating vestibular ganglia near the utricle and saccule. This substantial alteration of gene expression changes the organization of the apical part of the cochlea and results in the formation of ‘intragangliionic hair cells’. Our data and those of others suggest the following interaction of *Neurod1* with *Neurog1, Atoh1, Sox2* and *Fgf8* to regulate inner ear cellular identity (B, B’). We propose that after early and transient activation of *Neurod1* by *Neurog1* and *Atoh1* to differentiate neuron, *Neurod1* suppresses *Neurog1* to inhibit precursor proliferation and *Atoh1* to inhibit hair cell differentiation in neurons. These three way interactions result in formation of neurons with delayed hair cell differentiation. Cross inhibition of *Neurog1* and *Atoh1* was previously suggested [17, 20] and we suggest that *Neurod1* is a key intermediary player. *Neurod1* also regulates other cell fate determining genes like *Sox2* and *Fgf8* which may more directly related to the observed cell fate switch. We suggest that *Neurod1* deletion in early embryos disrupts this gene network and, as a consequence, the coordinated sequential neurosensory development of inner ear resulting in the transformation of some surviving neurons into ‘intraganglionic hair cells’ and alteration of the cell type specific differentiation of outer hair cells in the cochlea.

*Neurod1* regulates several genes involved in hair cell differentiation. *Atoh1*, *Pou4f3*, *Fgf8* and *Nhlh1* are prematurely expressed in the apical half of the cochlea in *Neurod1* CKO mutants (Fig. 9A,A’) and appear in hair cells within the sensory ganglia of the ear. There is also a transient change in *Sox2* expression and in *Neurog1* expression. Our results are best compatible with a suggestion that *Neurod1* expression in neurosensory precursors suppresses specific downstream genes (*Atoh1*, *Pou4f3*, *Nhlh1*, *Fgf8, Sox2*) necessary for general neurosensory and specific hair cell differentiation (Fig. 9B,B’). For example, the upregulation of *Fgf8* in some ‘outer hair cells’, which may change their fate to ‘inner hair cells’, suggest a more specific function of *Neurod1* in regulation of *Fgf8*. The effect of *Neurod1* on *Neurog1* is likely due to a direct, intracellular feedback loop (Fig. 9B,B’) and is in line with previous reports of such a feedback loop in olfactory receptor cell development [69]. In contrast, the effect of *Neurod1* on *Atoh1* expression could be either directly in the same cell as in the cerebellum [26] or could be mediated through an intermediary such as *Fgf8*, *Sox2* or an as yet to be determined factor within or between cells. Further analysis of other developing systems in which *Neurod1* and *Atoh1* are sequentially expressed or co-expressed, such as the dorsal cochlear nucleus [70] or the enteroendocrine intestine cells [71], are needed to establish generality of this feedback loop beyond the ear and the cerebellum.

Cross-regulation of *Neurog1* and *Atoh1* have been proposed for the spinal cord [72] and the mammalian ear [20] in which hair cells are massively reduced in *Neurog1* null mice [14]. However, in none of these cases has the interaction been directly demonstrated at the cellular or molecular level. Our data on the effect of *Neurod1* CKO mutants suggests that *Neurod1* is at least in the ear an intermediate factor that mediates such cross-inhibitory interactions between *Neurog1* and *Atoh1* (Fig. 9B, B’). The differences in effects of either loss of *Neurog1* or *Neurod1* on overall hair cell formation and specific hair cell developmental changes suggests that other downstream factors specific to *Neurog1* or *Neurod1* must exist that also mediate such cross-inhibitory interactions. *Fgf8* and possibly *Sox2* seem to be appropriate candidates to play this role. Further analysis of expression of these and other genes now identified as being changed in *Neurod1* CKO mice are needed for *Neurog1* null mice to fully understand the complexity of interaction of the genes in this developing system.

In summary, we propose that inner ear development resembles other developing systems insofar as sophisticated interactions of bHLH genes determine neuronal fate [3], [73]. In cooperation with other genes known to be expressed in the developing sensory neurons and hair cells [3], [4], [5], [74], [75], *Neurod1* may achieve neuronal differentiation not only through upregulation of appropriate downstream target genes [62] but also through suppression of other bHLH genes that mediate other states of cellular differentiation such as *Neurog1* [69] or *Atoh1* [26]. In the absence of *Neurod1*, several cell fate determining genes that would normally be suppressed are prematurely or continuously expressed (Figs 4,9). These expression changes result most likely in a cell fate change of neurosensory precursors into topologically inappropriate cells such as ‘intraganglionic hair cells’ and ‘ectopic inner hair cells’ (Fig. 9B’). Through some of the 500 genes directly regulated by *Neurod1* [62], *Neurod1* may actually mediate the cross-regulation of *Neurog1* and *Atoh1* as recently suggested [20]. Our findings show a more refined action of *Neurod1* in the developing ear than the previously suggested simple effects on neuronal survival and differentiation [15], [16], [29]. *Neurod1* may interact with other genes expressed during neurosensory development [36], likely mimicking better described systems in the complexity of their interaction [76] particularly at the promoter level [77]. Fully understanding this interplay is necessary to allow, through regulation of the levels of expression of *Neurod1*, the generation of ‘intraganglionic hair cells’ in deaf patients. Such ‘intraganglionic hair cells’ could imitate regular hair cells and sustain long-term cochlear implant function by maintaining viable neurons.

## Supporting Information

Figure S1 Atoh1 in situ hybridization shows a progressive base-to-apex reduction in wild-type cochlea (A, A’, A”) with faded expression in IHC and outermost OHCs. In contrast, Atoh1 remains uniformly expressed throughout the cochlea in Neurod1 CKO mice (B, B’, B”). Direct comparison of vestibular sensory epithelia shows that canal cristae are more reduced than utricle and saccule (C–F) which show mostly alterations in shape. Such qualitative changes are also apparent in anterior canal cristae where hair cells form in the non-sensory region of cruciate eminence in absence of Neurod1 (arrow in G,H). AC, anterior canal crista; HC, horizontal canal crista; S, saccule; U, utricle. Bar indicates 100 µm.
(0.63 MB TIF)

Figure S2 Our data suggest that Neurod1 specifies neurosensory precursors by refining cellular identity during early embryonic stage. We confirm this assumption studying the delayed knock out of Neurod1 using Tg (Atoh1cre), a CKO mutation that results in massive cerebellar defects (26). The in situ hybridizations of Atoh1 and Fgf8 show normal organization of inner ear with four rows of hair cells throughout the cochlea (A–B). We conclude that once Atoh1 has regulated its downstream target genes to specify the hair cell precursor's fate, later loss of Neurod1 cannot alter hair cell differentiation. IHC, inner hair cell; OHC, outer hair cell. Bar indicates 100 µm.
(0.52 MB TIF)
